# Impact of UV sterilization and short term storage on the *in vitro* release kinetics and bioactivity of biomolecules from electrospun scaffolds

**DOI:** 10.1038/s41598-019-51513-1

**Published:** 2019-10-22

**Authors:** Olivera Evrova, Damian Kellenberger, Chiara Scalera, Maurizio Calcagni, Pietro Giovanoli, Viola Vogel, Johanna Buschmann

**Affiliations:** 1 Division of Plastic Surgery and Hand Surgery, University Hospital Zurich, Sternwartstrasse 14, 8091 Zurich, Switzerland; 2 Laboratory of Applied Mechanobiology, ETH Zürich, Vladimir-Prelog-Weg 1-5/10, 8093 Zurich, Switzerland; 3 ab medica, via J. F. Kennedy, 10/12 - 20023 Cerro Maggiore (MI), Cerro Maggiore, Italy

**Keywords:** Preclinical research, Translational research

## Abstract

To effectively translate bioactive scaffolds into a preclinical setting, proper sterilization techniques and storage conditions need to be carefully considered, as the chosen sterilization technique and storage condition might affect the structural and mechanical properties of the scaffolds, as well as the bioactivity and release kinetics of the incorporated biomolecules. Since rarely tested or quantified, we show here in a proof-of-concept study how these parameters are affected by UV sterilization and one week storage at different temperatures using bioactive electrospun DegraPol scaffolds that were specifically designed for application in the field of tendon rupture repair. Even though UV sterilization and the different storage conditions did not impact the morphology or the physicochemical properties of the bioactive scaffolds, UV sterilization caused significant attenuation of the growth factor release kinetics, here platelet derived growth factor (PDGF-BB) release (by approx. 85%) and slight decrease in ascorbic acid release (by approx. 20%). In contrast, 4 °C and −20 °C storage did not have a major effect on the release kinetics of PDGF-BB, while storage at room temperature caused increase in PDGF-BB released. All storage conditions had little effect on ascorbic acid release. Equally important, neither UV sterilization nor storage affected the bioactivity of the released PDGF-BB, suggesting stability of the bioactive scaffolds for at least one week and showing potential for bioactive DegraPol scaffolds to be translated into an off-the-shelf available product. These parameters are expected to be scaffold and protein-dependent.

## Introduction

In the field of tissue engineering and regenerative medicine, bioactive materials and scaffolds are developed in multiple fashions for different applications. Among the many options and strategies, delivery of growth factors or other biomolecules from different scaffolds has been widely used to enhance and promote healing of different injuries and defects^1–3^. In comparison to only biomaterial scaffolds, development of bioactive scaffolds with growth factors or other biomolecules is more complicated since care should be taken not only for the stability, structural and mechanical properties of the scaffold, but also for maintaining bioactivity and appropriate release kinetics of the incorporated biomolecule. During the preclinical development of bioactive scaffolds envisioned for preclinical *in vitro* and *in vivo* studies and eventual clinical application, proper sterilization methods, as well as appropriate storage conditions for off-the-shelf availability are essential to be taken into consideration. Up to date, there are limited numbers of reports that provide information on the effects of storage and sterilization on bioactive scaffolds^4,5^. Thus, it is essential to develop standard protocols to quantify how sterilization and storage conditions affect the scaffold properties, as well as the bioactivity and the release kinetics of incorporated biomolecules.

One type of bioactive scaffolds widely employed in the field of tissue engineering are electrospun scaffolds with incorporated biomolecules within the fibers^6–13^. Bioactive electrospun scaffolds can be obtained by either aseptic production or by applying terminal sterilization, which is the preferred practice. For *in vitro* preclinical research, UV sterilization is the preferred and widely used sterilization method^14^. In contrast, commonly used sterilization methods for clinical applications include ethylene oxide (EtO) and γ-radiation^14^. So far, research has been performed to determine the effect of different sterilization methods (ethylene oxide, γ-radiation, UV) on the structural or mechanical properties of different electrospun scaffolds^14–18^ or other biomaterials^19^, but there are not many reports on the stability of bioactive electrospun scaffolds incorporating growth factors or other biomolecules, upon sterilization or different storage conditions^19–21^. Guided by careful quantifications, not only the material properties should be taken into account, but also the stability of the incorporated biomolecule and potential changes in its release profile when choosing the sterilization technique as well as the duration and temperature of storage.

Here in a proof-of-concept study, we quantified how sterilization and storage parameters are affecting the material properties, the bioactivity and the release kinetics of a growth factor and another small biomolecule. Previously in our work, we have developed an elastic, biocompatible and bioactive scaffold made from a polyester urethane block copolymer called DegraPol (DP), to be applied for tendon rupture repair^22,23^. The DP scaffold in a form of electrospun tube was shown to reduce adhesion formation in healing tendons^24^ and later was developed as a bioactive scaffold with incorporated growth factor (PDGF-BB) in order to promote tendon regeneration^25^. To further develop this or other bioactive scaffolds to be used in preclinical *in vivo* studies, sterilization and storage of the bioactive scaffolds would be crucial factors to be examined.

Hence, we explored the effects of UV sterilization and storage at different temperatures of the bioactive scaffolds with incorporated platelet-derived growth factor-BB (PDGF-BB) or ascorbic acid (as a further model biomolecule) and produced by emulsion electrospinning. Morphology and physicochemical properties of UV treated scaffolds or stored scaffolds at room temperature, 4 °C or −20 °C were compared to non-treated scaffolds. Moreover, the release kinetics of PDGF-BB and ascorbic acid and later the bioactivity of the released PDGF-BB from the sterilized and stored scaffolds were studied. These results would give an initial idea of how the release profile or bioactivity of molecules incorporated in electrospun scaffolds might be affected by different storage conditions and would aid in future design and development of other bioactive scaffolds.

## Results and Discussion

### Effect of UV sterilization and storage conditions on the chemical stability of the bioactive DP scaffolds

**Figure 1:**
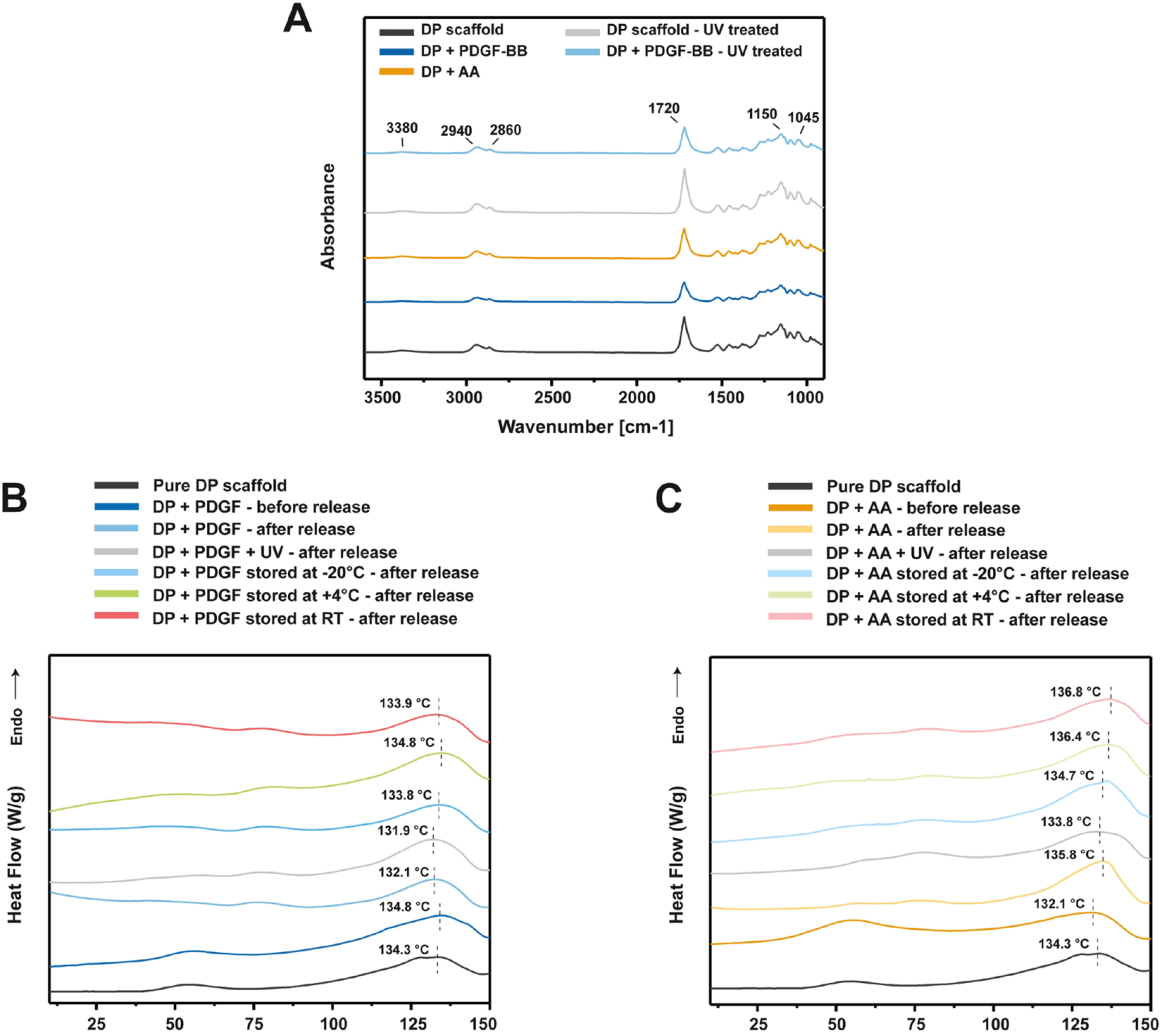
FTIR and DSC characterization of differently stored and UV sterilized bioactive DP scaffolds. (**A**) FTIR absorbance spectra for bioactive DP scaffolds incorporating PDGF-BB or ascorbic acid, with or without UV sterilization. (**B**,**C**) DSC thermograms (not to absolute scale) of first heating for non-stored, stored and UV sterilized bioactive DP scaffolds with PDGF-BB or ascorbic acid, after 30 days long *in vitro* release study, compared to pure DP scaffolds and bioactive scaffolds before release study.

To obtain insight on whether DP scaffolds/tubes can serve as off-the-shelf bioactive scaffolds for preclinical *in vitro* and *in vivo* studies, experiments to assess their stability upon storage at different temperature for a short period or UV sterilization were performed. To assess whether the DP polymer and the scaffolds undergo some changes associated with the electrospinning process or upon storage and UV sterilization, the different scaffolds were analyzed with Fourier Transformed Infrared Spectroscopy (FTIR) and Differential Scanning Calorimetry (DSC). Incorporation of PDGF-BB and ascorbic acid via emulsion electrospinning did not lead to any changes in the FTIR spectra when compared to pure DP scaffolds (Fig. 1A). UV sterilization of pure DP scaffold or DP scaffold with PDGF-BB did not lead to any changes visible in the FTIR spectra when compared to non-sterilized pure DP scaffolds or non-sterilized scaffolds with PDGF-BB. In the FTIR spectra of all scaffolds a big band at 1720 cm^−1^ is present and can be assigned to the carbonyl group (C=O stretch) in the ester bonds of DP, since it is a polyester urethane copolymer^25,26^. The bands in the region of 1300 to 1000 cm^−1^, specifically 1150 cm^−1^, can be attributed to the symmetric and asymmetric vibrations of C-O bonds in esters. The bands at 2940 cm^−1^ and 2860 cm^−1^ are due to the stretching vibrations of the –CH2 and –CH3 groups^27^, while the small absorption band at 3380 cm^−1^ corresponds to the stretching of NH groups in the DP structure. Since no new absorption peaks are visible in the spectra of the differently treated scaffolds, it can be concluded that no new chemical bond is formed between the growth factor or ascorbic acid and the polymer, even in the UV sterilized scaffolds, so the individual components were not chemically modified. In other studies, it was also observed that UV sterilization did not lead to visible changes in the FTIR spectra of other types of materials/scaffolds, like PLA^28^, polyurethanes^29^ or PLGA^30^.

DSC analysis was used to assess the thermal properties of DP scaffolds with PDGF-BB or ascorbic acid that have undergone storage or UV sterilization, before and after 30 days *in vitro* release period. Figure 1B,C show that sterilized emulsion electrospun scaffolds with PDGF-BB or ascorbic acid after 30 days *in vitro* release period, exhibit similar thermal behavior as scaffolds which were not sterilized, before and after 30 days release period. Similarly, the scaffolds which were stored for 1 week and underwent 30 days release period *in vitro*, had similar thermal behavior as the non-stored DP scaffolds. The melting temperatures (T_m_) of each scaffold were in a narrow range of 131 °C to 137 °C, confirming that the T_m_ of DP scaffolds is not significantly affected upon the electrospinning process, UV sterilization, storage and *in vitro* release, and are within the reported range of T_m_ for different DP types^31^. Additionally, the data shows that within the 30 days release period, the differently stored DP scaffolds did not undergo a major degradation process which could have affected the thermal properties of the scaffolds.

**Figure 2:**
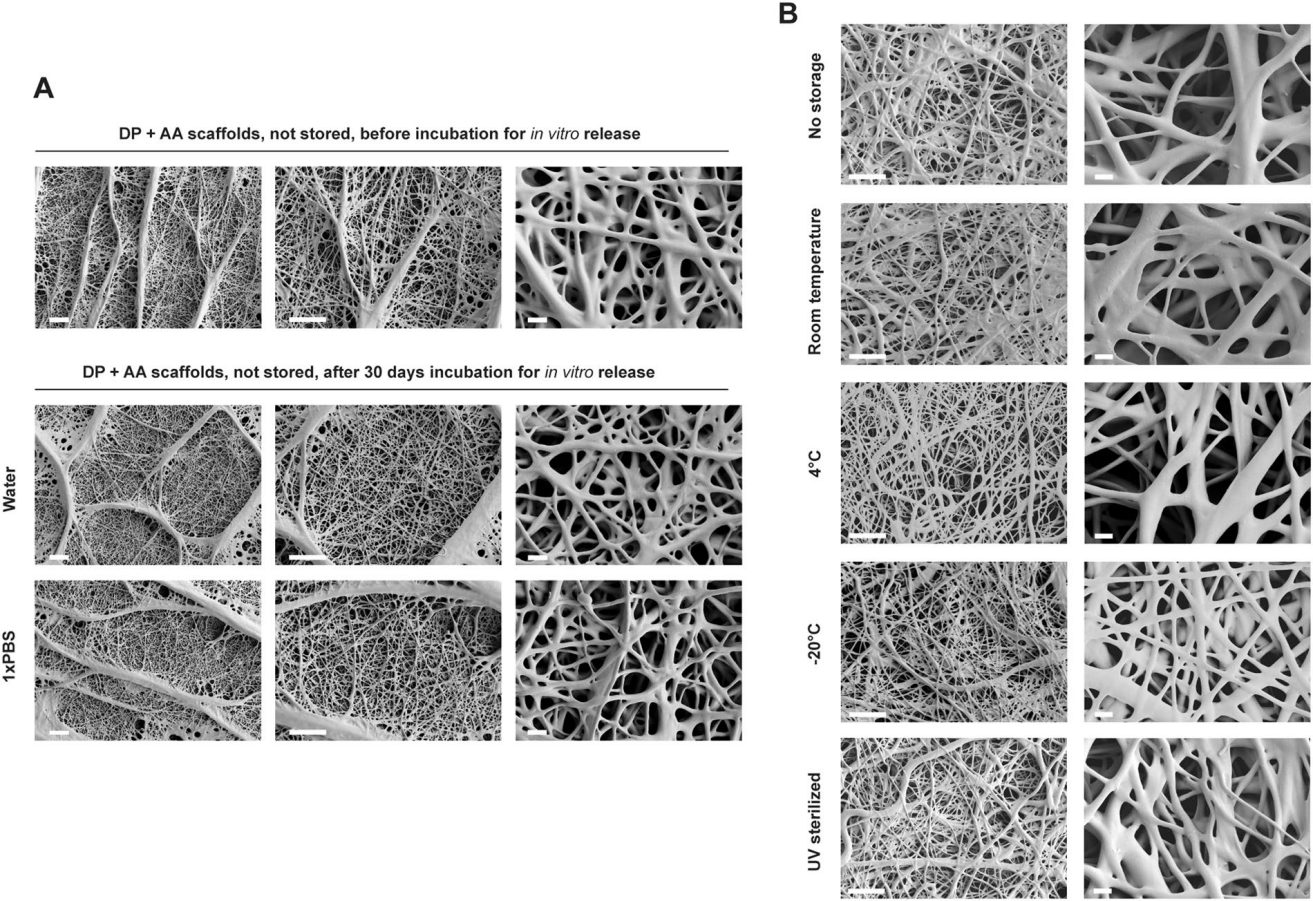
Scaffold morphology of non-stored and stored bioactive DP scaffolds. (**A**) SEM micrographs of non stored DP scaffolds with incorporated ascorbic acid, before or after 30 days *in vitro* release in water or 1xPBS. (**B**) SEM micrographs of non-stored and differently stored DP scaffolds with incorporated ascorbic acid, after 14 days *in vitro* release in water. Scale bars: 40 μm (lower magnification) and 4 μm (higher magnification).

The fiber morphology of non-treated, non-stored DP scaffolds with incorporated ascorbic acid, before an *in vitro* release period and after 30 days *in vitro* release in water or 1xPBS was assessed by SEM (Fig. 2A). Additionally, differently stored or UV treated DP scaffolds that have undergone subsequent 14 days *in vitro* release period were imaged (Fig. 2B). The SEM micrographs obtained show that there are no changes on the fiber surface before and after the release period (either after 14 or 30 days). Similarly, there were no visible differences in morphology between non-stored scaffolds and stored scaffolds, or after UV sterilization. No visible degradation or pore formation was observed on the DP fiber surface in any of the conditions. These results suggest that after storage of 1 week at different conditions and subsequent *in vitro* release period, the scaffolds do not undergo a visible morphological change which could suggest difference in the release profile of molecules from them. One week of storage is generally accepted to be a long enough period between the production (electrospinning) and the *in vitro* application or the implantation into a preclinical animal model. Moreover, no visible morphological differences were observed upon 30 days release period in water (used as release medium for ascorbic acid) or 1xPBS pH 7.4 (used as release medium for PDGF-BB). Since the studied DP has a degradation half-life of 3 months *in vivo*^24^, it was expected that no morphological changes like pore formation or degradation would be readily visible after 30 days release period under *in vitro* conditions. However, depending on the polymer type or scaffold design, morphological changes like fiber degradation due to faster polymer degradation, or pore formation onto the fibers as part of fiber architecture^32,33^ or due to soluble component dissolving away from the fibers (e.g. PEG)^30,34,35^, can significantly impact the stability and release kinetics of the bioactive scaffolds after UV sterilization or short term storage.

### Release kinetics of ascorbic acid and PDGF-BB from UV sterilized and differently stored bioactive DP scaffolds

**Figure 3:**
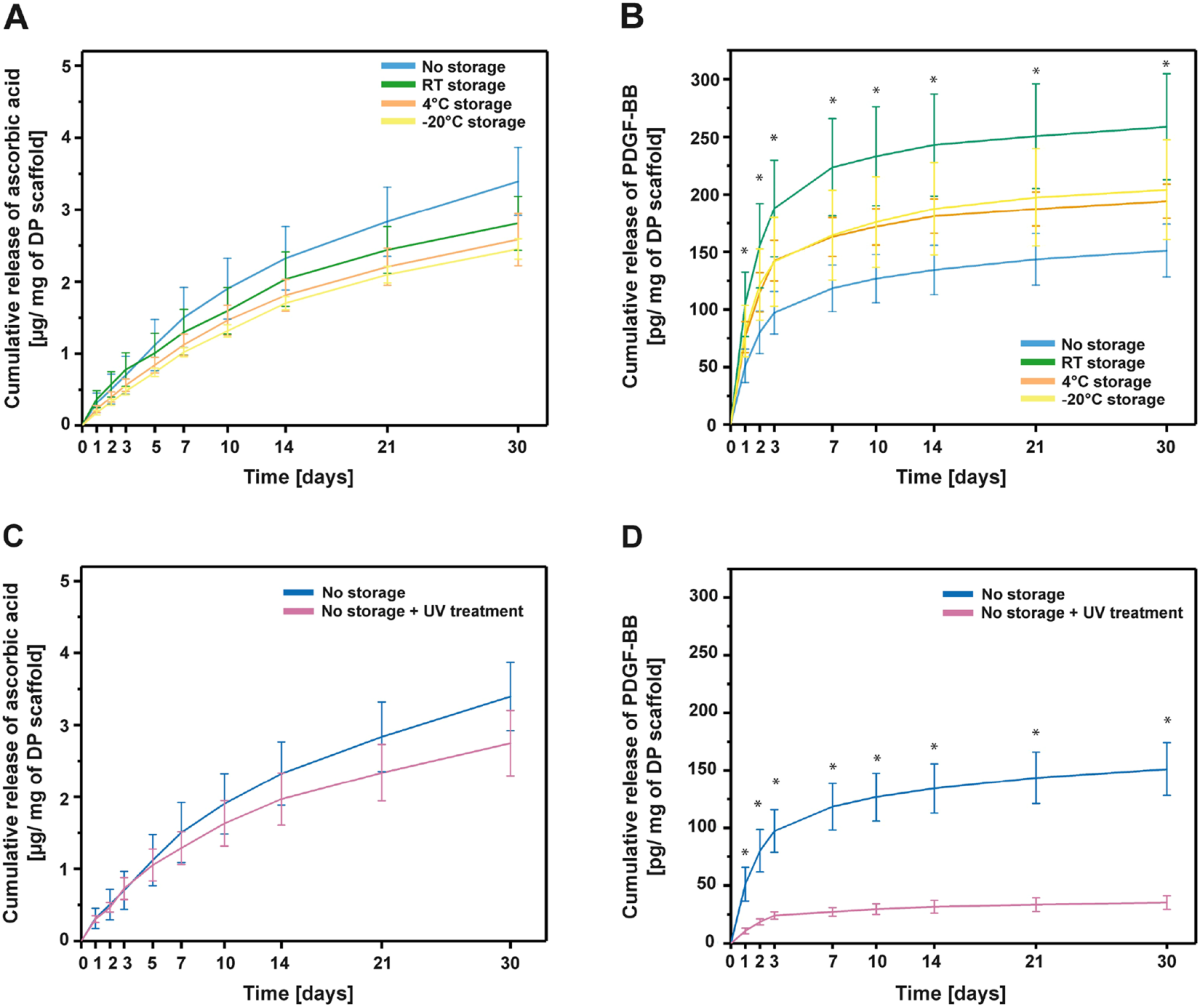
PDGF-BB and ascorbic acid release from differently stored and UV sterilized bioactive DP scaffolds. (**A**,**B**) *In vitro* cumulative release of ascorbic acid and PDGF-BB from differently stored (not stored, room temperature/+4 °C/−20 °C stored) emulsion electrospun DP scaffolds, respectively. (**C**,**D**) *In vitro* cumulative release of ascorbic acid and PDGF-BB from non-sterilized or UV sterilized emulsion electrospun DP scaffolds, respectively. *p < 0.05.

One of the crucial parameters for bioactive scaffolds is the release profile of the incorporated molecules within the scaffolds. It would be desirable to have bioactive scaffolds as off-the-shelf products that retain their release kinetics and bioactive properties as after production. For this purpose, the stability of the bioactive scaffolds and the incorporated molecules over certain period of time and environmental conditions is a crucial parameter. Moreover, any scaffold used preclinically must be properly sterilized, without the risk of causing an infection. Taking these points into consideration, we studied the short term stability of emulsion electrospun DP scaffolds with incorporated PDGF-BB or ascorbic acid, and the release profile upon 1 week storage period at different conditions. Additionally, we assessed the release profile of UV sterilized scaffolds, since UV sterilization is a commonly used and readily available method used for *in vitro* research. The release of ascorbic acid from scaffolds stored for 1 week at room temperature (RT), 4 °C or −20 °C is shown in Fig. 3A. All of the scaffolds showed a gradual, sustained release profile in water, and the highest amount of ascorbic acid released per mg of scaffold was observed for the non-stored scaffolds. Scaffolds stored at RT, 4 °C or −20 °C showed the same release profile, with slight decrease in the overall ascorbic acid released. However, no significant differences were observed due to storage when compared to the non-stored scaffolds. Similarly, the release of PDGF-BB from differently stored scaffolds was assessed (Fig. 3B). All of the scaffolds showed a sustained release profile of PDGF-BB. Contrary to the release profile of ascorbic acid, the highest overall release of PDGF-BB per mg of scaffold was observed for the RT stored scaffolds, while the lowest overall release for the non-stored DP scaffolds. The increase in cumulative release of PDGF-BB from RT stored scaffolds was significantly higher when compared to the one from non-stored scaffolds. Scaffolds stored at 4 °C or −20 °C showed very similar cumulative release and without significant differences when compared to RT stored and non-stored scaffolds.

These results show different trends for the effect of storage on the release of PDGF-BB or ascorbic acid. In the storage period, DP scaffolds might undergo small changes in the scaffolds structure (initial minimal degradation, polymer aging), which might be happening faster under room temperature conditions, when compared to 4 °C or −20 °C conditions. Due to these initial changes (not observed in the scaffold morphology), the release profile of molecules incorporated within the fibers might change, with an increase in release, since the release is diffusion based and depended on how closely the molecules are to the fiber surface. On the other hand, not only the stability and changes in DP scaffolds might affect the release profile measured, but also the stability of the incorporated molecule. Depending on how stable the incorporated molecule is overtime, the measured quantities of the same might change. Thus, the different trend of ascorbic acid release from stored scaffolds might be due to a decreased stability of incorporated ascorbic acid, which has degraded overtime and was not quantified during the measurement. Based only on the release data, the stability of PDGF-BB does not seem to be affected due to different storage conditions.

The release of ascorbic acid and PDGF-BB was also assessed from UV sterilized DP scaffolds (Fig. 3C,D). With UV sterilization (30 min), both types of scaffolds showed decreased cumulative amount of molecule released per mg of scaffold, but the release of PDGF-BB was majorly impacted by UV sterilization (decreased by approximately 85%) (Fig. 3D). UV sterilization did not impact the release of ascorbic acid significantly, and only a slight decreased in release was observed (decreased by approximately 20%) (Fig. 3C).

We hypothesize that this can be attributed to changes in polymer surface chemistry after UV treatment upon which PDGF-BB molecules adsorb onto the fibers more strongly and are not released; or it can be due to deleterious effect of UV light on PDGF-BB causing degradation of growth factor close to the fiber surface, and only small part protected inside the fibers to be detected once released^36^. Visible changes on FTIR spectra post UV treatment (Fig. 1A) which might suggest changes in bonds, affecting PDGF-BB release, were not observed. Similarly, no morphological changes like fiber fusion or fiber melting were observed on SEM micrographs (Fig. 2B) nor on FTIR spectra of the bioactive scaffolds (Fig. 1A), which could have affected the PDGF-BB release post UV treatment.

Similar effect of attenuated growth factor release upon UV treatment was observed for TGFβ3 release from PLGA microspheres. However, also visible surface changes were observed on the PLGA microspheres upon UV treatment, which could have contributed to the decreased release^37^. Changes in degradation, molecular weight or morphology upon UV treatment (1 hour) were also reported for PLGA and P(LLA-CL) nanofibers^38^. Contrary, other studies reported no visible differences in morphology of PLGA microspheres, where UV treatment was performed for only 10 minutes^39^ or after 2 hours UV treatment of poly(d,l-lactic acid)–poly(ethylene glycol)–monomethyl ether diblock copolymers (Me.PEG–PLA)^40^. Thus, discrepancy in these results and others reported in literature^41^ regarding effects of UV treatment of different scaffolds, suggest that optimal treatment conditions may vary for different materials and should be explored and optimized for different delivery systems. Moreover, since UV sterilization is routinely used for *in vitro* experiments involving bioactive scaffolds, results point to the fact that care needs to be taken when UV sterilization is performed on scaffolds later used for cell seeding, proliferation and other types of *in vitro* assays, since the release or bioactivity of biomolecules might differ from the ones observed from non-sterilized scaffolds. The same is true for UV sterilized scaffolds intended to be applied in preclinical animal models.

Our data suggests decrease in release kinetics upon UV treatment of bioactive scaffolds, but it has to be emphasized that the release behavior of any other biomolecule upon UV sterilization may differ from the one observed for this specific pair of scaffold/biomolecule. Depending on the interaction between the two components and on their individual constitution, release kinetics and UV impact on release kinetics might differ from our model system here.

### Bioactivity of released PDGF-BB from UV sterilized or differently stored bioactive DP scaffolds

**Figure 4:**
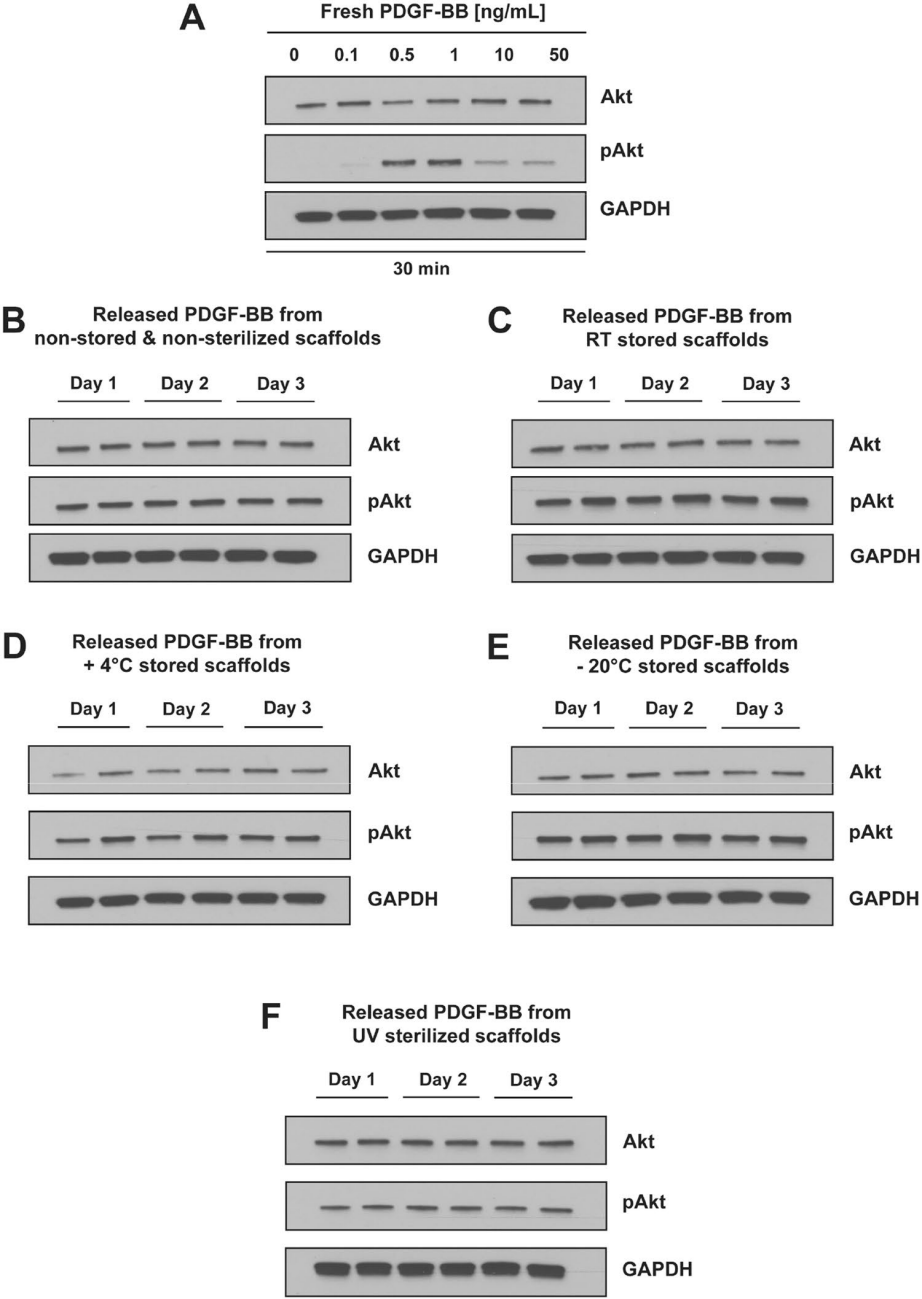
Bioactivity of released PDGF-BB from differently stored and UV sterilized bioactive DP scaffolds by Akt activation. (**A**) The phosphorylation status of Akt (pAkt) to dose-dependent PDGF-BB (0, 0.1, 0.5, 1, 10 and 50 ng/mL) supplementation (cells treated for 30 min) was analyzed by western blot. Presence of band for pAkt signifies phosphorylation of Akt and with that the ability of PDGF-BB to activate the PDGFR/PI3K/Akt pathway. The phosphorylation status of Akt (pAkt) to PDGF-BB released from (**B**) non-stored and non-sterilized DP scaffolds, (**C**) scaffolds stored for 1 week at room temperature (RT) or D) 4 °C or (**E**) −20 °C or (**F**) not stored but UV sterilized for 30 min. The release was performed in serum-free medium with 0.1% BSA and release samples for Day 1, 2 and 3 were tested (in duplicates), in order to obtain initial idea whether bioactivity of PDGF-BB is preserved after the specific treatments. Activation of Akt signaling pathway by released PDGF-BB, thus leading to increase in pAkt, shows retained bioactivity of the growth factor after the specific storage time period or the UV sterilization. For uncropped images, we refer to Fig. S1.

Not only the release kinetics can be affected by the sterilization or storage of bioactive scaffolds, but more importantly the bioactivity of the biomolecules incorporated within the scaffolds can be impacted. In the case of sensitive molecules like growth factors, it is essential to determine whether any processing step in the production of the bioactive scaffold impacts their bioactivity. In this study, we decided to assess only the bioactivity of the released PDGF-BB after the different treatments in order to assess their impact on the bioactive scaffolds. The biologically active form of PDGF-BB can bind to tyrosine kinase receptors on the cell surface, trigger dimerization and activation of the receptors and subsequent downstream cell signaling pathways^42^. From the several signaling pathways which can be activated, the well characterized PDGFR/PI3K/Akt pathway was chosen for determining the bioactivity of the released PDGF-BB^43^. In this regard, the phosphorylation of Akt (pAkt) at a serine residue (S473) was tested on serum-starved rabbit tenocytes after stimulation with samples containing released PDGF-BB from differently stored or UV sterilized scaffolds. As a positive control, fresh samples of PDGF-BB with known concentrations (0.1, 0.5, 1, 10 and 50 ng/mL) were used for stimulating serum starved tenocytes, for a period of 30 min. At the time point of 30 minutes stimulation, the signal of pAkt increased within the range of 0.1–1 ng/mL PDGF-BB, followed by a decrease at higher PDGF-BB concentrations (10 and 50 ng/mL). (Figs 4A and S1). This allowed to establish a baseline of pAkt levels at different concentrations of fresh, bioactive PDGF-BB in stimulated serum-starved tenocytes, which could be used as a standard curve to determine bioactivity of the released PDGF-BB from the differently treated scaffolds. As amounts of released PDGF-BB were never >2.5 ng/mL for each time point (Fig. S2), retained bioactivity of released samples was expected to yield a pAkt signal similar to 1 ng/mL fresh PDGF-BB, with slight variation dependent on the effective PDGF-BB concentration in the sample. Released PDGF-BB from the non-stored or differently stored DP scaffolds was determined to be bioactive in all of the release samples tested for Day 1, 2 and 3, visible by the presence of a signal for pAkt. There are no visible differences between the PDGF-BB released from non-stored or stored DP scaffolds. This confirms that the bioactive DP scaffolds are stable and can be stored for at least 7 days under different storage temperatures and still the scaffolds and the PDGF-BB incorporated would retain their stability and bioactivity. Released PDGF-BB from the sterilized DP scaffolds over Day 1, 2 and 3 was also determined to be bioactive, as it triggered phosphorylation of Akt (Fig. 4F). The signal for pAkt in the samples from UV sterilized scaffolds is not as strong as for the non-sterilized samples (Fig. 4B) or stored samples (Fig. 4C,D,E), but this can be attributed to the fact that the concentration of the PDGF-BB in these samples is lower, as shown by the released PDGF-BB amounts presented in Fig. 3D. Additionally, bioactivity assay performed by monitoring cell proliferation of cells seeded directly onto UV sterilized bioactive DP scaffolds releasing PDGF-BB showed increased proliferation when compared to cells seeded onto DP scaffolds without PDGF-BB, suggesting that on/and near the scaffold enough amount of PDGF-BB retained bioactivity (Fig. S3) and thus complementing the results observed in Fig. 4F ^25^. Since the PDGF-BB released from the UV sterilized scaffolds was bioactive, it offers the possibility to use UV sterilization of bioactive scaffolds if needed and desired, even though optimal duration might be necessary to be determined for each material/scaffolds differently.

## Summary and Conclusion

While UV sterilization (30 min) had no impact on the physicochemical characteristics or morphology of the bioactive electrospun DP scaffolds, it impacted the release of PDGF-BB by significantly decreasing the cumulative PDGF-BB amount released over a period of 30 days. Also the storage temperature (room temperature, 4 °C or −20 °C) affected the biomolecule release, with the effect much more pronounced for the growth factor PDGF-BB than for ascorbic acid. Significantly though, both sterilization and the 1-week storage did not impact the bioactivity of the incorporated and released PDGF-BB which was tested by its ability to activate the PDGFR/PI3K/Akt pathway and was shown to be bioactive upon release.

In conclusion, these data are promising as they show the potential for bioactive DegraPol scaffolds to be translated into an off-the-shelf available product. It is expected though that each of the parameters tested here depends on the material properties, how it is processed into a scaffold and on the biochemical and biophysical properties of the drug that is released. Not only is the chemical composition of the molecules a crucial factor to be considered with respect to hydrolysis or other degradation processes, but also the interaction between the (polymer) scaffold and the biomolecule in terms of Van der Waals forces, covalent bonding etc. Quantifying how UV sterilization and storage affects bioactive scaffolds is crucial prior to designing preclinical studies with bioactive scaffolds for diverse tissue engineering applications. It should be pointed out that the sterilization and storage of another scaffold/biomolecule combination might be more significantly affected than what is observed here for bioactive DP scaffolds. Moreover, the effect of combination of storage and sterilization subsequently might not be additive. Our objective here is to show the importance of evaluating these important factors during fabrication and pre-clinical applications of any bioactive scaffold.

## Experimental Procedures

### Materials

A biodegradable polyester urethane block copolymer, commercially available as DegraPol15 (DP) (M_w_ = 65 kDa) was kindly provided Ab Medica, Italy. The polymer was produced according to the procedure described earlier^25,44^. Chloroform, ≥99.8% (0.5–1.0% ethanol as stabilized), 1,1,1,3,3,3-hexafluoro-2-propanol (HFP) and L-ascorbic acid 2-phosphate sesquimagnesium salt hydrate, ≥95%, RPMI vitamins solution, RIPA buffer, phosphatase inhibitors and 0.5 M EDTA solution were purchased from Sigma-Aldrich, Switzerland. Recombinant human PDGF-BB and PDGF-BB ELISA kit were purchased from PeproTech. Ham’s F12 cell culture media, gentamicin, amphotericin B and Fetal Bovine Serum (FBS) were bought from Biowest, while non-essential amino acids solution, Pierce micro BCA protein assay and Pierce ECL Western Blotting Substrate were purchased from Thermo Scientific. Primaria^TM^ tissue culture plates were purchased from Corning. Primary antibodies used for western blot included rabbit anti Akt (9272 S, Cell Signaling Technology), rabbit anti pAkt (Ser473) (4060 S, Cell Signaling Technology) and rabbit anti GAPDH (G9545, Sigma Aldrich). Donkey anti rabbit antibody conjugated with HRP (Jackson) was used as secondary antibody for western blot. 4–20% gradient polyacrylamide gels were purchased from Bio-Rad, Switzerland.

### Incorporation of PDGF-BB and ascorbic acid into DP polymer solutions

DegraPol polymer solutions of 12 wt% were prepared by dissolving the polymer overnight at room temperature in a mixture of chloroform/HFP (80:20 wt/wt). For emulsion preparation before electrospinning, PDGF-BB was diluted into 0.1% bovine serum albumin (BSA) in MilliQ water at a concentration of 40 μg/mL, while ascorbic acid was dissolved in MilliQ water at a concentration of 50 mg/mL. For water in-oil emulsions, 200 μL of PDGF-BB or AA stock solution were added drop-wise to 5 g of 12 wt% DP polymer solution while stirring at 500 rpm for 2 min. Afterwards, the emulsions were sonicated with a probe ultrasonicator (Sonoplus HD 2070, Bandelin, Germany) for 2 min at 50% amplitude. Immediately afterwards, the emulsion was used for electrospinning.

### Scaffold production by emulsion electrospinning

For emulsion electrospinning, in-house assembled electrospinning device was used, consisting of a spinning head with a blunt end made of stainless steel tube (1 mm inner diameter and 0.3 mm wall thickness, Angst & Pfister AG, Zürich Switzerland), a DC high voltage supply (Glassman High Voltage Inc., High Birdge, NJ, USA), hollow cylindrical rotating aluminum mandrel as a collector and a syringe pump (SP210cZ, WPI, Germany). The prepared DP polymer emulsion was loaded into 2 mL syringe (B. Braun, Melsungen AG, Germany) and pumped into the spinning head. During emulsion electrospinning, 1 mL/h flow rate, 12.5 kV applied voltage and 15 cm working distance were used. Electrospinning was performed at room temperature (22–24 °C) and less than 35% humidity.

### Sterilization and storage of bioactive emulsion electrospun DP scaffolds

Bioactive emulsion electrospun scaffolds with incorporated PDGF-BB or ascorbic acid were produced with emulsion electrospinning and were dried overnight under vacuum. Afterwards, scaffolds were either stored under different conditions or directly used for subsequent experiments. For the storage experiments, scaffolds were enclosed in plastic dishes and stored for a period of 1 week at room temperature, +4 °C or −20 °C. UV sterilization of 30 min was performed to dry, non-stored scaffolds and the sterilized scaffolds were used directly for further experiments.

### Scanning electron microscopy (SEM)

Before and after respective storage, sterilization or release experiments, small pieces of the scaffolds were mounted on metal stubs with conductive double-sided tape. The samples were sputter coated (SCD 500, Bal-tec) with 10 nm platinum coating and then examined by SEM (Zeiss SUPRA 50 VP, Zeiss, Cambridge, UK), at an accelerating voltage of 5 kV.

### Fourier-transform infrared spectroscopy (FTIR) characterization

Fourier transform infrared spectroscopy was carried out on a Cary 670 Fourier transform infrared (FTIR) spectrometer (Agilent Technologies) equipped with a SPECAC attenuated total reflection (ATR) diamond accessory. Dry, emulsion electrospun DP scaffolds not sterilized or UV sterilized, without and with incorporated PDGF-BB or ascorbic acid were characterized with FTIR. Spectra were collected within the wavenumber range of 500–4000 cm^−1^ and the interferometer was scanned with an acquisition rate of 37.5 kHz at 2 cm^−1^ resolution. A total of 64 scans were averaged to obtain one spectrum, whereas three regions of the scaffold per condition were scanned. The spectral data was pre-processed by applying rubber band baseline correction in Python. For the presentation of the results, normalization was performed to the C=O peak at 1720 cm^−1^.

### Thermal analysis (DSC) of DP scaffolds

Thermal analysis was performed using a differential scanning calorimeter, DSC 822e (Mettler Toledo, Griefensee, Switzerland), calibrated with indium standards. Emulsion electrospun DP scaffolds loaded with PDGF-BB or ascorbic acid, UV sterilized or not sterilized were analyzed. Scaffolds which were stored under the various conditions and which have undergone an *in vitro* release experiment for 30 days, were also analyzed. DSC thermograms were recorded under a nitrogen flow, by heating the samples from −10 °C to 150 °C, with heating and cooling rate of 10 °C/min. Two heating cycles were performed with an intermittent cooling cycle. To determine the properties of the actual scaffolds used throughout the study, only the first heating cycle was taken into consideration. The glass transition temperature of DegraPol was determined as the midpoint temperature of the glass transition event.

### Release of PDGF-BB from emulsion electrospun DP scaffolds

Emulsion electrospun DP scaffolds loaded with PDGF-BB were cut in small pieces (10–30 mg), shortly wetted in 50% ethanol and rinsed in MilliQ water. Scaffolds which were not stored, with or without UV sterilization, as well as stored scaffolds (n = 9) were placed in low protein binding microtubes and 500 μL of 0.1% BSA in 1xPBS were added as release medium. The samples were incubated at 37 °C and 5% CO_2_ under mild shaking. At respective time points (1, 2, 3, 7, 10, 14, 21, and 30 days), samples were taken out and placed in new microtubes and 500 μL of fresh release medium were added. The release samples collected were stored at −20 °C until further quantification. Released PDGF-BB was quantified with PDGF-BB ELISA kit according to the manufacturer’s protocol. The release of PDGF-BB is represented as cumulative release normalized to the weight of DP scaffolds as pg/mg of DP scaffold. Results are shown as mean ± standard deviation. Non stored UV sterilized scaffolds were compared with non-stored and non-sterilized scaffolds. Stored scaffolds were compared with non-stored scaffolds.

### Release of ascorbic acid from emulsion electrospun DP scaffolds

Emulsion electrospun DP scaffolds loaded with ascorbic acid were cut in small pieces (10–30 mg), shortly wetted in 50% ethanol and rinsed in MilliQ water. Scaffolds which were not stored, with or without UV sterilization, as well as stored scaffolds (n = 6) were placed in low protein binding microtubes and 500 μL of MilliQ water were added as release medium. The samples were incubated at 37 °C and 5% CO_2_ under mild shaking. At respective time points (1, 2, 3, 5, 7, 10, 14, 21, and 30 days), samples were taken out and placed in new microtubes and 500 μL of fresh MilliQ water were added, while the concentration of ascorbic acid was determined spectrophotometrically at 260 nm (Infinite200, Tecan Inc., Switzerland). The release of ascorbic acid is represented as cumulative release normalized to the weight of DP scaffolds as μg/mg of DP scaffold. Results are shown as mean ± standard deviation. Non stored UV sterilized scaffolds were compared with non-stored and non-sterilized scaffolds. Stored scaffolds were compared with non-stored scaffolds.

### Cell isolation and cell culture

To test whether the released PDGF-BB from the non-stored, stored and UV sterilized bioactive DP scaffolds is still bioactive, its ability to promote phosphorylation of Ser473 in the serine/threonine kinase Akt was tested. In order to test the bioactivity of released PDGF, rabbit tenocytes from Achilles tendons of New Zealand White rabbits were used as an *in vitro* model. Cells were isolated from the tendons using the migration method. Briefly, tendons were extracted and washed with Hank’s Balanced Salt Solution (1x HBSS with Ca^2+^ and Mg^2+^) with 200 μg/mL gentamicin and 2.5 μg/mL amphotericin B. The tendons were cleaned from the surrounding tissue, the central part of the tendons was cut into very small pieces (<2 mm) and they were washed three times in the 1x HBSS buffer. Multiple tissue pieces were placed into each tissue culture plate (Primaria^TM^) and a small amount of cell culture medium (Ham’s F12, 10% FBS, 200 µg/mL gentamicin and 2.5 µg/mL amphotericin B) was added onto each tissue piece. Tissues were allowed to attach onto the cell culture plates for 2 hours at 37 °C and 5% CO_2_ before adding 10 mL of cell culture media in each plate. The plates with the tissues were not moved for the first 5 days, to decrease tissue detachment upon plate movement and to allow cells to start migrating out from the tissues. First medium change was done after 5 days, and subsequently culture medium was changed every third day. After approximately 2 weeks, tissue pieces were removed from the plates, and cells were allowed to proliferate for 1 week more before cryopreservation. Cryopreserved rabbit tenocytes were thawed, resuspended in culture medium (Ham’s F12 with 10% FBS and 50 µg/mL gentamicin) and cultured at 37 °C and 5% CO_2_ with media being changed every second day. Tenocytes at passage 3 were used for the experiments.

All experiments were carried out in accordance with relevant Swiss regulations and guidelines. Moreover, the University Hospital Zurich approved all experimental protocols.

### Bioactivity of released PDGF-BB from differently treated DP scaffolds

In order to determine whether the released PDGF-BB from stored, non-stored or UV sterilized scaffolds is still bioactive, its ability to promote phosphorylation of the serine/threonine kinase Akt was tested. Scaffolds were stored or UV sterilized and put for release in serum-free medium (Ham’s F12, 1x RPMI vitamins solution, 1x non-essential amino acids solution, 50 µg/mL gentamicin) with 0.1% BSA. Release samples (1.5 mL total volume) were taken at respective time points over a period of 3 days (Day 1, 2 and 3) and stored at −20 °C until further use. Rabbit tenocytes cultured in complete medium (Ham’s F12 with 10% FBS and 50 μg/mL gentamicin) were seeded into 6-well plates (TPP, Switzerland), with a density of 150 000 cells/well. The following day, the culture medium was exchanged for a serum-free medium (Ham’s F12, 1x non-essential amino acids solution, 1xRPMI vitamins solution and 50 μg/mL gentamicin) and cells were starved overnight. Afterwards, the serum-free medium was replaced with the respective release samples collected earlier and cells were incubated for 30 minutes before cell lysis was performed. A fresh PDGF-BB with known concentrations (0.1, 0.5, 1, 10 and 50 ng/mL in serum free medium) were used as positive controls, while serum-free medium without any supplements served as a negative control. After 30 minutes incubation, whole cell lysate extracts were collected by scraping the cells on ice using 100 μL of RIPA buffer combined with protease and phosphatase inhibitors and 5 mM EDTA. Collected lysates were incubated on an end-over-end shaker for 20 minutes at 4 °C and then centrifuged at 12,000 x g for 15 min to remove cell debris. The total protein content of the samples was measured by the Pierce micro BCA protein assay. For performing the Western blots, equal amounts of protein per sample (10 μg) were incubated with loading buffer (5x protein loading buffer with 100 mM dithithreitol) at 95 °C for 10 min before loading them onto the gel. Protein samples were run on 4–20% gradient polyacrylamide gels.

### Statistical analysis

The data were analyzed with Origin (OriginLab, Northampton, MA). Values are expressed as means ± standard deviations. One-way analysis of variance (one-way ANOVA) was performed to test the differences between groups in all the experiments, using comparison post hoc test (Bonferroni) for significance. *p* values of less than 0.05 were considered statistically significant and are indicated with an asterisk within graphs (*p < 0.05, **p < 0.01, ***p < 0.001).

## Supplementary information

Supplementary Information
