# Supplementary material for: Impact of UV sterilization and short term storage on the in vitro release kinetics and bioactivity of biomolecules from electrospun scaffolds
Source: Sci Rep. 2019 Oct 22;9:15117. doi: 10.1038/s41598-019-51513-1 (PMC6805903; doi:10.1038/s41598-019-51513-1)
Supplement: Supplementary file 1 — Supplementary Information [file 41598_2019_51513_MOESM1_ESM.pdf]

Supplementary Information

Impact of UV sterilization and short term storage on the in vitro release kinetics and bioactivity of biomolecules from electrospun scaffolds

Olivera Evrova[1,2], *Damian Kellenberger*[1], *Chiara Scalera*[3], *Maurizio Calcagni*[1], *Pietro Giovanoli*[1], *Viola Vogel*[2] *and Johanna Buschmann*[1]*

[1] Division of Plastic Surgery and Hand Surgery, University Hospital Zurich, Sternwartstrasse 14, 8091 Zurich, Switzerland

[2] Laboratory of Applied Mechanobiology, ETH Zürich, Vladimir-Prelog-Weg 1-5/10, 8093 Zurich, Switzerland

[3] ab medica, via J. F. Kennedy 10/12, 20023 Cerro Maggiore (MI), Italy

Figure 4A

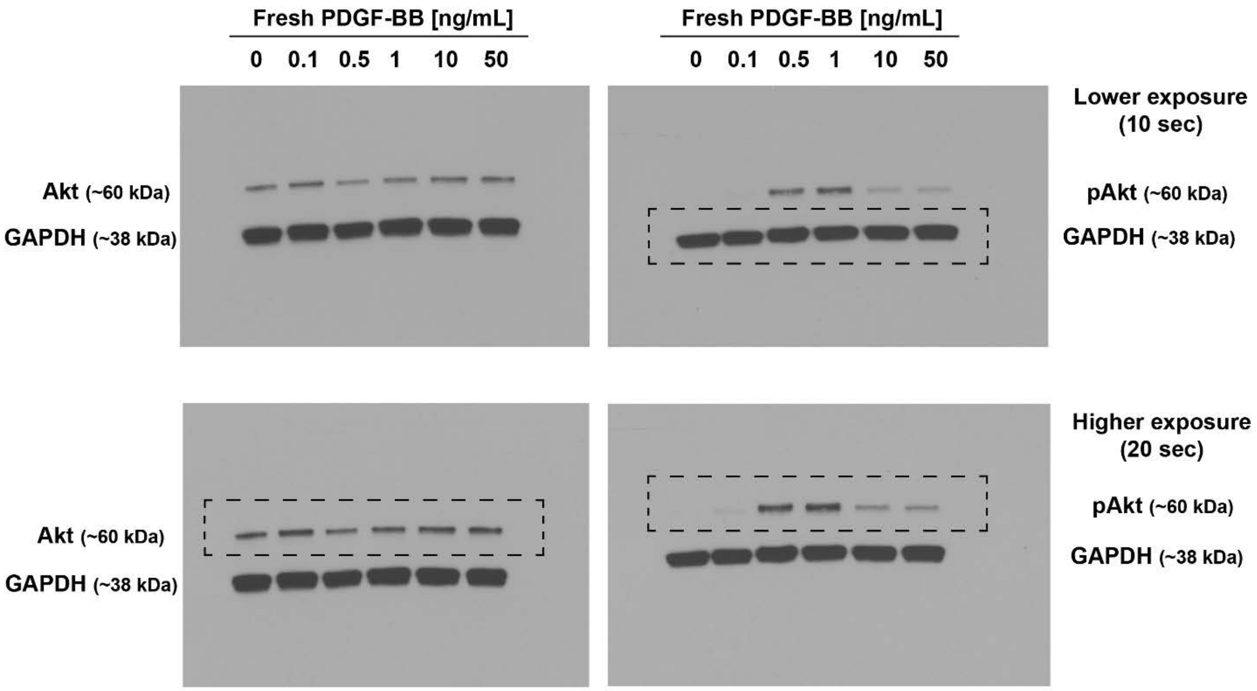

Figure 4B

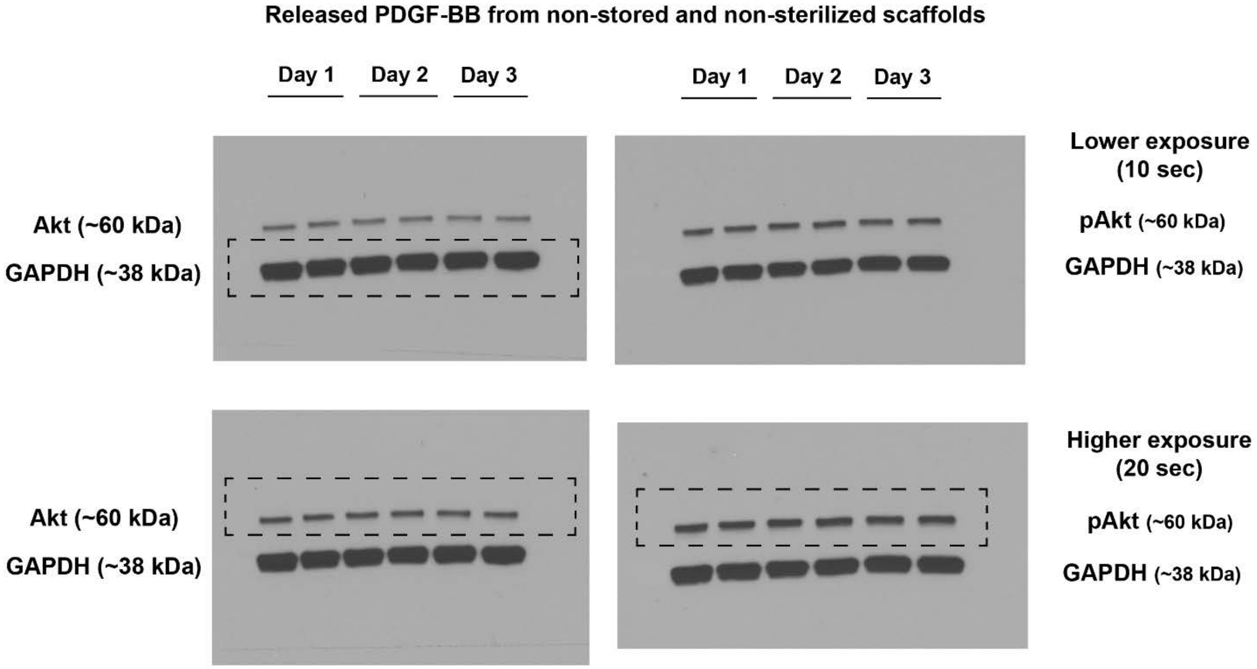

Figure 4C

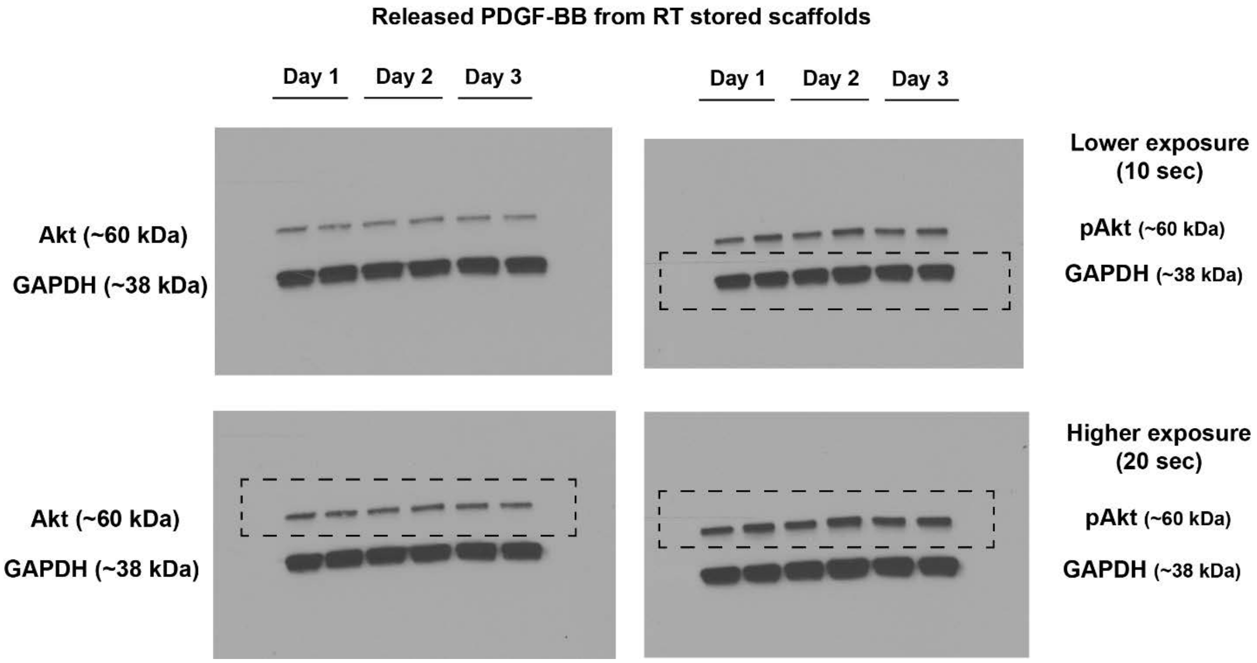

Figure 4D

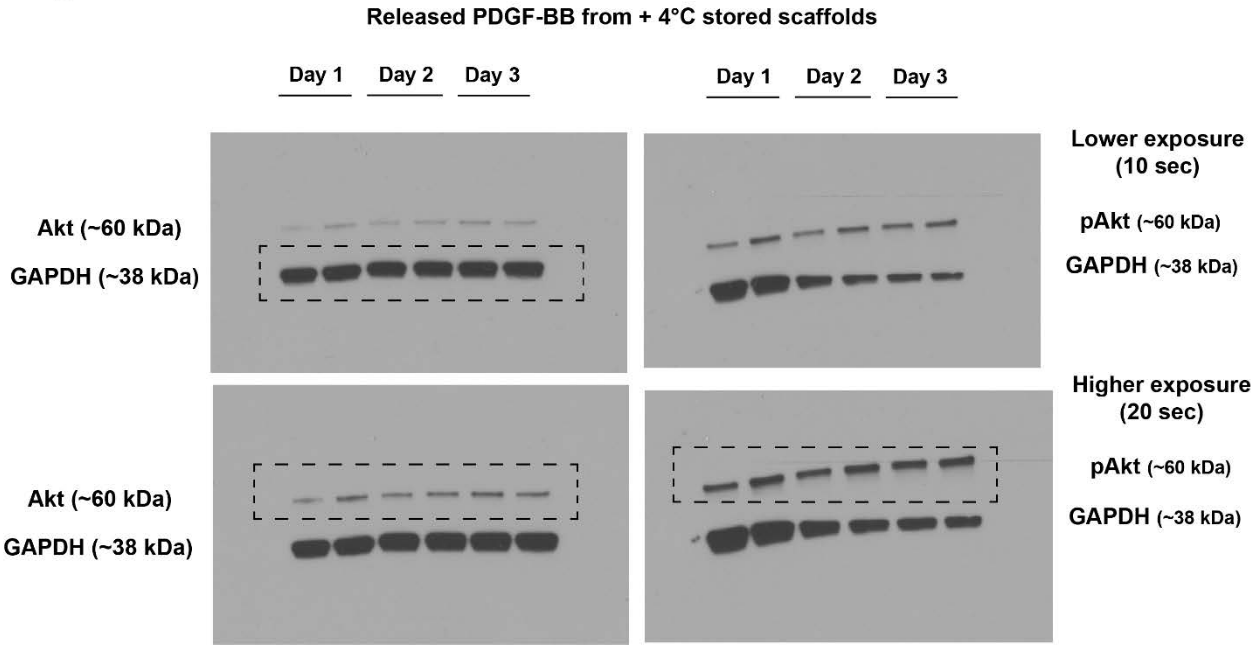

Figure 4F

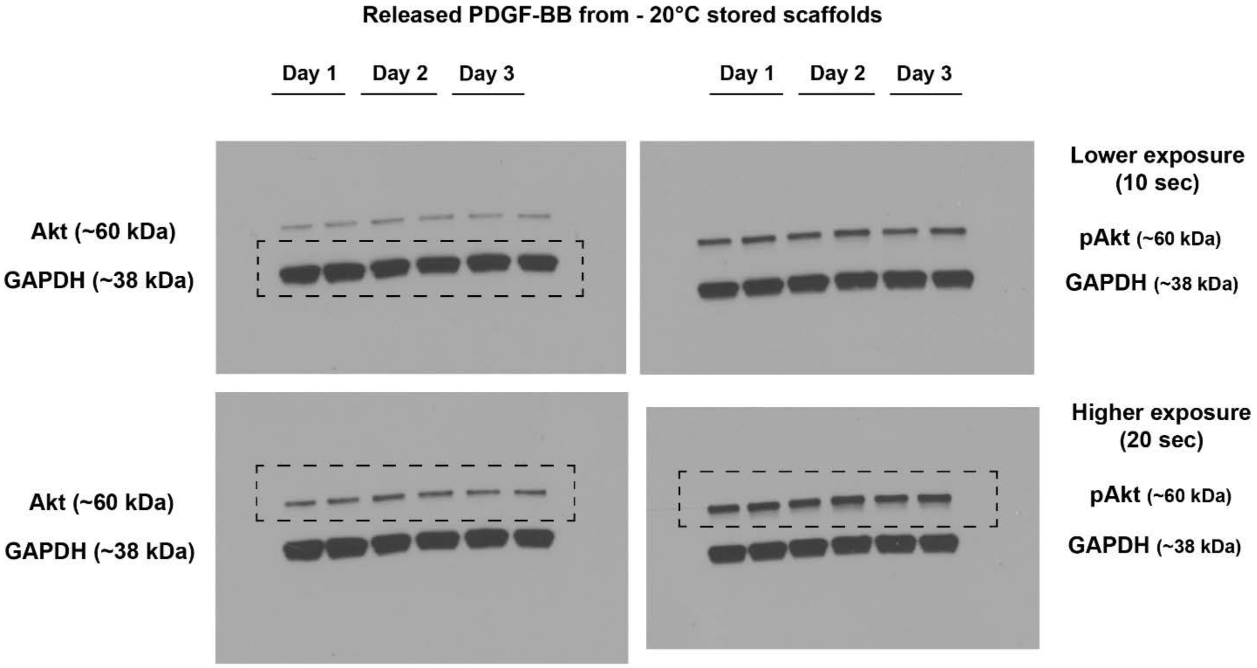

Figure S1. Uncropped Western blot images. These images refer to figures displayed in the main manuscript **Figure 4**.

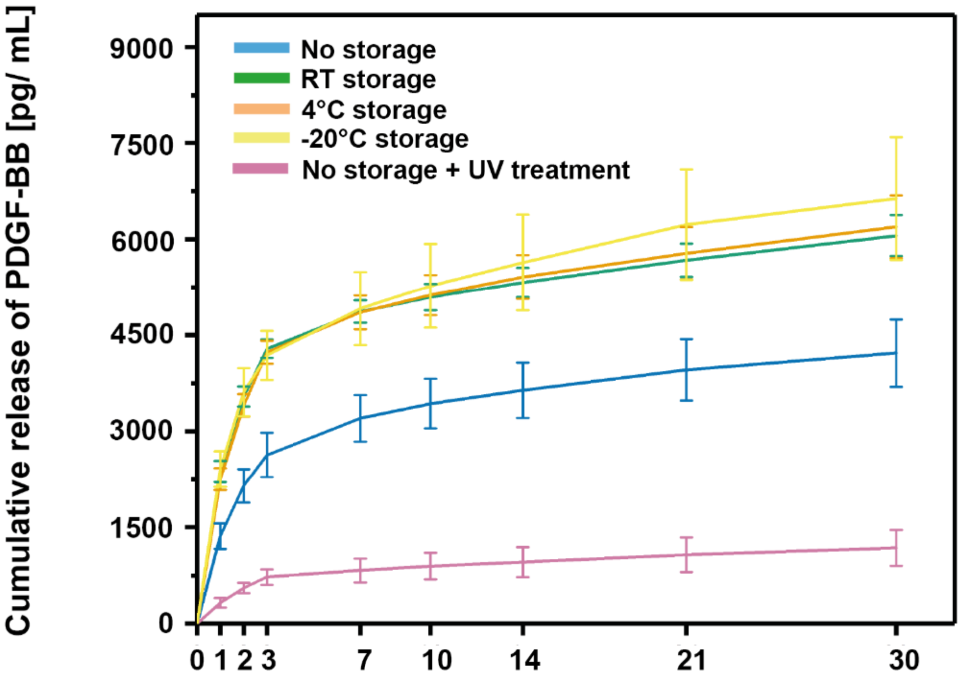

Figure S2. Cumulative release of PDGF-BB [pg/ mL] from differently stored and UV sterilized bioactive DP scaffolds. *In vitro* cumulative release of PDGF-BB from differently stored (not stored, room temperature/ +4°C/ -20°C stored/ stored + UV treatment) emulsion electrospun DP scaffolds, respectively. The data shown here corresponds to the data presented in **Figure 3B** and **D**, but without being normalized to the total weight of DP scaffolds, showing typical concentrations of PDGF-BB obtained during release experiments.

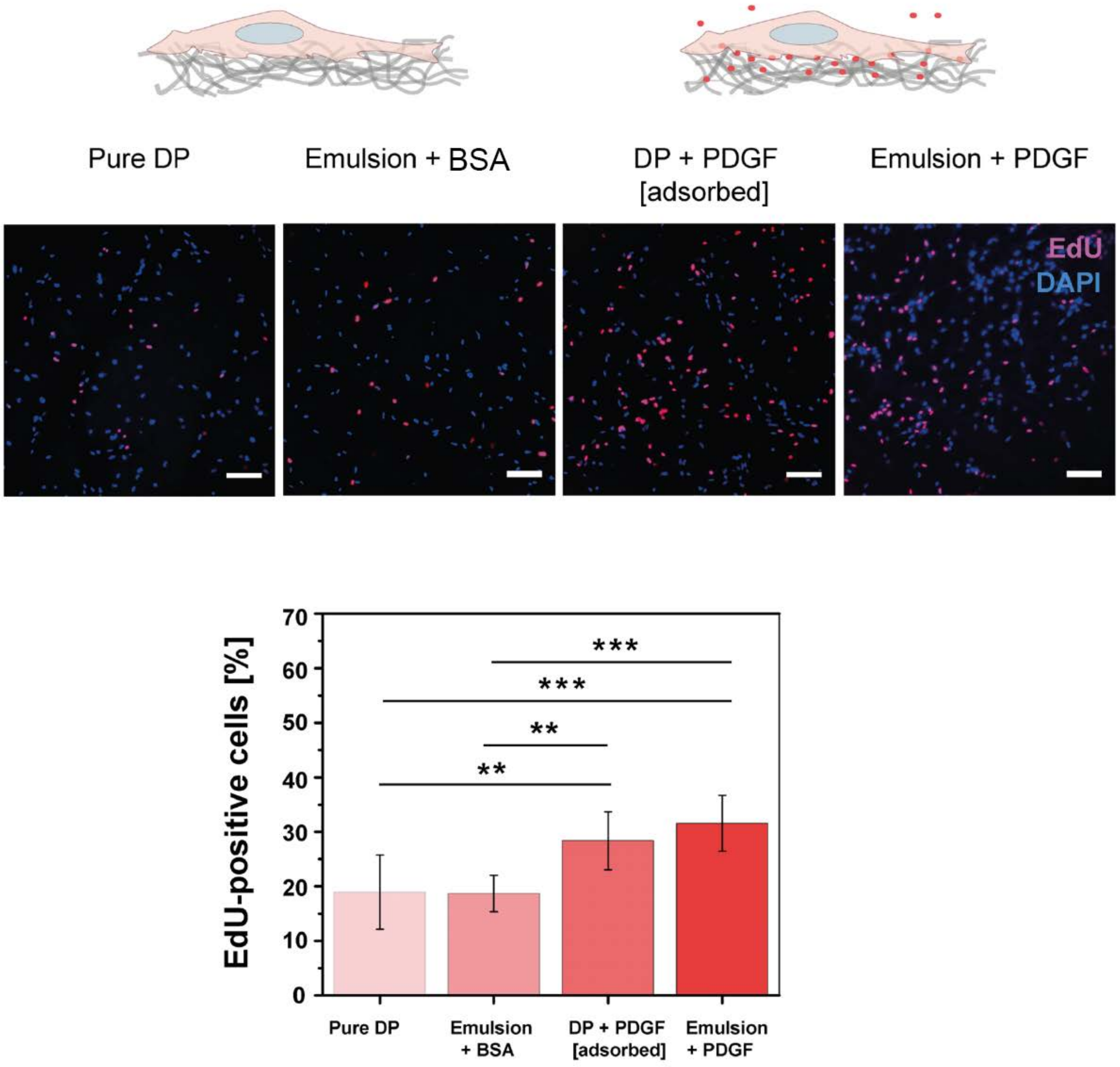

Figure S3. Bioactivity of incorporated PDGF-BB on UV sterilized DP scaffolds (Emulsion + PDGF-BB), under serum^{+} conditions, expressed as EdU-positive cells [%] and in comparison with physically adsorbed PDGF-BB on DP scaffolds (DP + PDGF [adsorbed]), emulsion DP scaffold containing only rabbit serum albumin (Emulsion + BSA) or pure DP scaffold (** $p < 0.01$, *** $p < 0.001$.). Figure has been adapted from[1] with copyright permission obtained from *Macromolecular Bioscience*. Scale bar: 100 µm.

References used in the Supplementary Information

1. Evrova, O. *et al.* Bioactive, Elastic, and Biodegradable Emulsion Electrospun DegraPol Tube Delivering PDGF-BB for Tendon Rupture Repair. *Macromolecular bioscience* **16**, 1048-1063 (2016).
